# A novel pathogenic classification of cancers

**DOI:** 10.1186/s12935-014-0113-9

**Published:** 2014-11-30

**Authors:** Carlos Sonnenschein, Barbara Davis, Ana M Soto

**Affiliations:** Department of Integrative Physiology and Pathobiology, Tufts University School of Medicine, 136 Harrison Avenue, Boston, MA 02111 USA

**Keywords:** Carcinogenesis, Pathobiology, Metastasis, Neoplasia, Mutation, Tissue organization field, Cancer theories

## Abstract

According to contemporary epidemiological and experimental evidence, we propose a novel classification of cancers based on pathogenesis instead of classifications based on histological appearance of cancer. This new scheme first defines cancers as either **1.*inborn errors of development*** or **2.*sporadic*** ones, and then sub-defines the former into **1A. inborn inherited errors of development**, being those due to mutations contributed by one or both parents’ gametes to the developing conceptus, and **1B. inborn induced errors of development** when the malformations and/or cancers are due to environmental carcinogenic exposure during pregnancy. It is anticipated that the origin of an increasing number of so-called sporadic cancers will turn out to be linked to the **inborn induced errors of development** group.

## Introduction

Human cancers have been classified according to diverse criteria primarily to serve the needs of the medical profession to diagnose, stage, prognosticate and treat the disease. ^a^For these pragmatic purposes, pathological classifications of cancer are based on organ of origin of the tumor and predominant cell type (epithelial or mesenchymal), with a long list of sub-classifications including (but not limited to) whether the tumor is benign or malignant (e.g., adenoma vs. carcinoma; fibroma vs. sarcoma), encapsulated or invasive, whether it contains components of different cell types or tissues (e.g., “desmoplastic adenocarcinoma”), and whether it displays low or high proliferation rates (mitotic indices). In recent years, a major effort has been devoted to characterize tumors based on their molecular features, particularly referring to genetic mutations and/or patterns of gene expression. Prognostic and therapeutic advances based on molecular features included, for example, identifying the estrogen and progesterone receptor and HER2/Neu status of breast cancers, or the mutational status of gastric stromal sarcomas and response to Gleevec [1]. The correlation between genomic somatic mutations in cells of “sporadic” cancers with a specific targeted therapy has been interpreted as being causal. However, according to recent pronouncements by the very proponents of such theoretical links, successes have been disappointing [2–5]. Admittedly, cancers are complex biological phenomena whose full understanding remains incomplete [3,6].

Regardless of whether carcinogenesis represents either a cell-based [3] or a tissue-based [6,7] phenomenon, from an etiopathogenic perspective, two main types of cancers are apparent: they can either be *inherited* or “sporadic”. *Inherited* cancers refer to those cancers that have an obvious or suspected link to germ-line mutations present in chromosomes that are passed on from one generation to the next. *Sporadic* cancers refer to those cancers that are assumed to lack an obvious inherited component; instead, it has been proposed that they are the result of the life-long accumulation of spontaneous or induced mutations in a single “normal” cell. Again, this classification does not address whether the cancer process is initiated within a cell as Boveri [8], Nowell [9] and most others have favored since the 20^th^ century, or instead, at the tissue level [10].

### Theories of carcinogenesis and metastases

There are divergent opinions regarding the level of biological organization at which cancer originates. The somatic mutation theory of carcinogenesis (SMT) defines cancer as a *cell-based* disease [8,9,11]. Its fundamental premise is that cancer is due to the accumulation of spontaneous or induced somatic mutations and/or chromosomal aberrations that alter the control of proliferation in a single cell that eventually will generate a tumor [8,12,13]. Boveri called this cell “the cancer cell” [8] and later it was renamed “the renegade cell” by Weinberg [14]. In this context, cancer becomes a clonal disease [9]. A seldom mentioned additional premise associated to SMT has been that *quiescence* rather than *proliferation* is the default state of cells in multicellular organisms [15–17]. Adoption of this latter premise implies that cells need to be stimulated directly in order to proliferate; following this rationale, since the 1950s, “growth factors” and, since the 1980s, oncogenes have been proposed as stimulators of cell proliferation [15,18].

From the second half of the 20th century onward, SMT gained standing due to staggering advances in genetics and molecular biology, and the de-emphasizing of physiology and developmental biology in cancer pathogenesis. The initial claim that cancers were due to a single mutation in a cell in culture conditions [19], referred to as malignant transformation, was followed by claims that the number of somatic mutations responsible for such a transformation in human tumors increased from single digits to the thousands [20,21]. Eventually, the SMT morphed from its original simplicity into the 6 “hallmarks of cancer” in 2000 [11], with 2 more being added a decade later [22].

In contrast to the SMT, the tissue organization field theory of carcinogenesis (TOFT) posits that cancer is i) a *tissue-based* disease, and that, explicitly, ii) *proliferation* is the default state of all cells [10]. That cancer is due to a pathological interaction between tissues is not a new claim; this was predicated by German pathologists during the second half of the 19th century [23,24]. The merits of both SMT and TOFT to explain carcinogenesis have been debated elsewhere based on experimental, clinical and theoretical grounds [6,7,25].

In the last decade, given the increasing lacks of fit between the premises of SMT and the evidence gathered from the huge amount of data generated by novel technical improvements in gene sequencing, a merging of the *cell-based* SMT and *tissue-based* components has been proposed as an add-on to the original SMT [11,18,26–28]. Based on grounds that SMT and TOFT are centered on a) different levels of biological organization (*cell* for SMT, *tissue* for TOFT) and b) opposite premises regarding the proliferative default state (*quiescence* for SMT and *proliferation* for TOFT), we and others have argued against accepting this compromise [6,7,10,29,30]. Moreover, in addition to evidence challenging the need for somatic mutations to significantly participate in the carcinogenic process [31], experimental and clinically-based evidence has documented that solid tumor carcinogenesis can occur in the absence of somatic mutations [32,33]. Also, equally robust experimental and clinical evidence shows that stromal alterations lead to neoplasia of the parenchyma as exemplified in leukemia [34–36].

As a result of the lacks of fit referred to above, another variant of SMT has been proposed, namely, the cancer stem cell (CSC) theory of carcinogenesis. Despite aggressive efforts directed at identifying normal stem cells and cancer stem cells, these entities remain as operational and rather elusive concepts [37–39]. In addition, the stem cell niche appears to be made up of epithelial cells plus the adjacent stroma; under this perspective, stemness is likely to be conferred by the niche and not by an autonomous epithelial cell-based property [37,40]. Thus, the CSC theory would represent another example of the type of “compromise” theory (SMT plus a tissue-based component) referred to above.

### A pathogenetic classification of cancers

**Figure 1:**
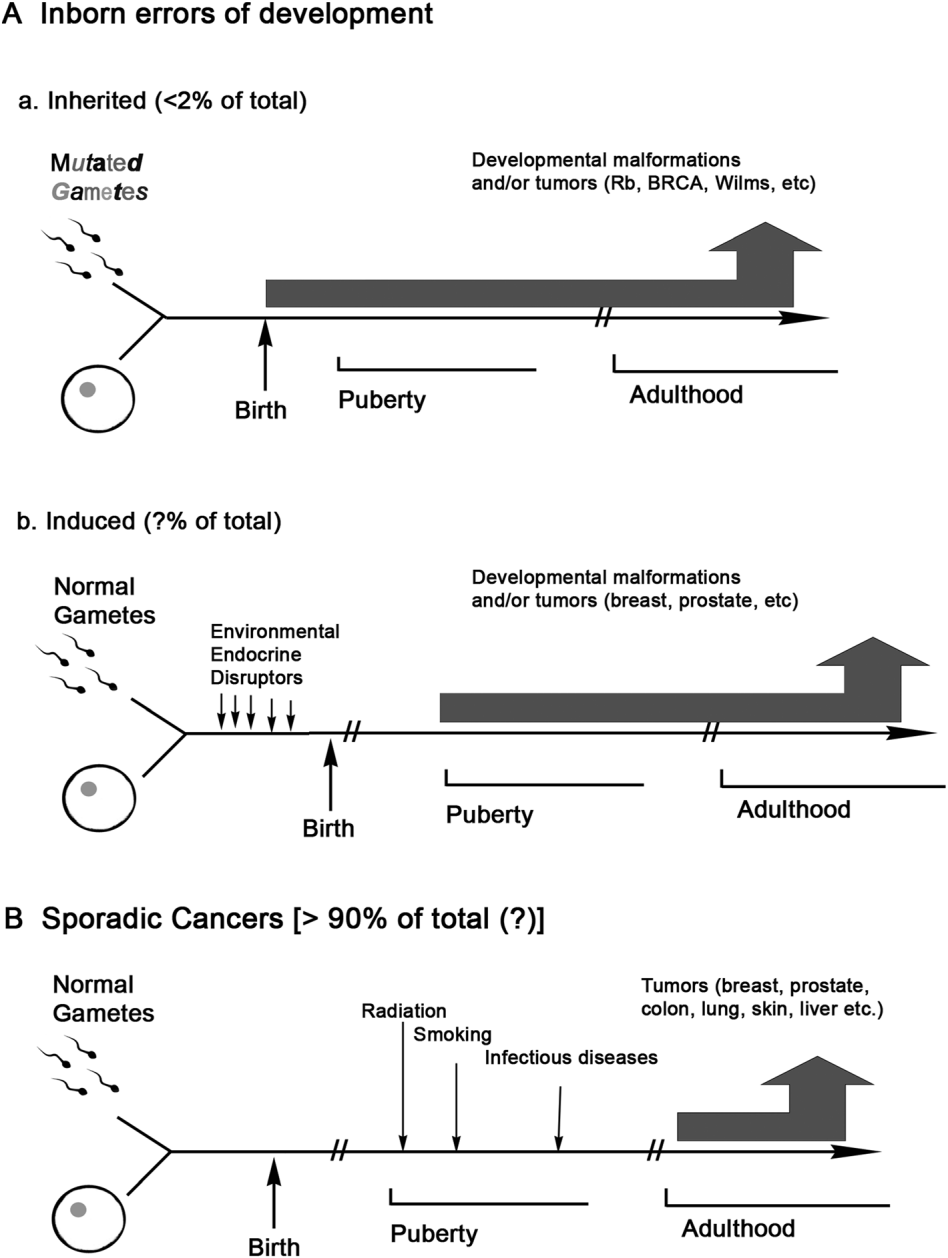
**Novel pathogenetic classification of neoplasia.** This classification distinguishes 2 main types of neoplasia as inborn errors of development **(A)** and sporadic cancers **(B)**. A is further classified into inborn errors of development that may be either inherited **(a)** or induced **(b)**.

In Figure 1, we outline our proposed novel etiopathogenic classification of cancers. It is comprised of two main groups of tumors, namely, **1. Inborn errors of development.** Within this group, we identify 2 sub-groups of neoplasms. First, there are **1A. Inborn*inherited*errors of development** - the result of a process initiated by a germ-line mutation(s) in the genome of one or both gametes (sperm and/or ovum). The mutated genome of the resulting zygote will endow all the cells in the morphogenetic fields of the developing organism with such a genomic mutation(s). The altered expression of these mutated genes could take place either during organogenesis, tissue remodeling and/or repair. This provides a temporal-dependent dimension to the carcinogenic process. Examples of this variety of inborn error of development include retinoblastoma, Gorlin syndrome, xeroderma pigmentosa, BRCA-1 and-2 neoplasia, and many others (for a comprehensive listing and description of these tumors see [41,42]).

Remarkably, King et al. [43] noticed in a cohort of patients carrying BRCA mutations that breast cancer risk by age 50 among carriers born before 1940 was 24%, whereas it was 67% among those born after 1940 [43]. These authors concluded that “non-genetic factors may significantly influence the penetrance even of high-penetrance mutations”. On the one hand, this evidence argues in favor of identifying those non-genetic environmental factors - presumably due to exposure to pollutants that significantly increase the cancer incidence not only of inborn inherited errors of development, but also of sporadic tumors. On the other hand, their observations strengthen the merits for postulating a novel pathogenetic classification of neoplasms that would reflect a significant participation of the environment in carcinogenesis.

Within the group of inborn errors of development, we identify a second subgroup of neoplasms that we named **1B. Inborn*induced*errors of development** (Figure 1). As the name implies, tumors and/or malformations would be due to alterations of the fetal environment, exemplified by exposure to environmental chemicals, such as synthetic hormones (Diethylstilbestrol (DES)) as well as by elevated levels of endogenous hormones [44–46]. Exposure of rodent embryos, fetuses and neonates to environmental estrogens generate premalignant and malignant neoplastic lesions in diverse tissues later in life [47,48]. In humans, exposure of fetuses to DES during the first trimester of pregnancy, correlated with the appearance of vaginal clear cell carcinomas during puberty and early adulthood. A conclusive correlation between exposure to DES and clear cell carcinoma was established because this rare neoplasm appeared in non-DES exposed populations only in post-menopausal women [49]. Animal experiments showing the development of adenosis, pre-neoplastic lesions considered precursors of clear cell carcinomas also suggests a causal link between a synthetic estrogen, DES, and the rare cancer described above [50]. Consistent with the effect of DES exposure in rats, there was an increased incidence of breast carcinomas in the above-mentioned cohort of women when they reached the prevalent age at which breast cancer most commonly occurs [51,52].

Furthermore, reports increasingly indicate that environmental chemicals are important causes in generating tumors. Among them, there are the environmental endocrine disruptors (EED) that are defined as an exogenous chemical, or mixture of chemicals, that interferes with any aspect of hormone action [53]. For example, Bisphenol-A (BPA) has been shown to increase the incidence of hormone-related cancers in rodents [46]. EEDs are suspected to be a factor in human breast and prostate cancers over the last 50 years [47,54]. Because BPA as well as other EEDs are not mutagens, the fact that they induce cancer cannot be adequately explained by the SMT, but is better understood from the TOFT perspective as due to faulty cell-cell and/or tissue-tissue interactions [54,55]. It is in this context that we consider cancer as “development gone awry”.

The World Health Organization’s cancer agency, IARC, has extrapolated industrialization with damaging lifestyle changes as also implying a correlation between direct and/or indirect effects of greater exposure in and out of the workplace (which includes pregnant women) to noxious chemicals and higher cancer incidence [56,57]. Since the 2008 estimates, breast cancer incidence has increased by more than 20% and mortality by 14%. Based on these statistics, IARC subsequently acknowledged the emergence of a “toxic epidemic” of cancers in developing nations [58].

**2. “Sporadic” tumors.** Neoplasms due to germ-line mutations have been considered to represent less than 5% of all cancers [42,59], whereas “sporadic” ones represent the vast majority of clinical cancers. The characterization of cancers as “sporadic” extends to any cancer that appears to have no *obvious* link to germ-line mutations (Figure 1). Their characteristics and properties have been described in great detail in the biomedical literature and will not be dealt with in this article. Our novel classification anticipates, however, that many of what are now considered as “sporadic” cancers will in the future be reclassified as **Inborn induced errors of development**. Briefly, the term “sporadic” cancers would now be restricted to those cancers that result from carcinogenic exposures after gestation.

## Conclusions

A pathogenic classification of cancer is being proposed based on novel insights on experimental carcinogenesis, and from evidence collected through highly sophisticated genome analysis in search of somatic mutations. This classification identifies the presence of 2 major groups of cancers, namely, 1) inborn errors of development, and 2) “sporadic” ones. Based on whether or not genomic mutations are responsible for the emergence of a cancer tissue phenotype, the former group is further classified into 1A) inborn inherited errors of development and 1B) inborn induced errors of development. This classification better reflects current views about how cancers develop, and anticipates that the incidence of “sporadic” cancers now being diagnosed will diminish by being reclassified as our type 1B.

## Endnote

^a^For the sake of simplicity, we will use the nouns cancers, neoplasms and tumors interchangeably.
